# Editorials

**Published:** 1911-09

**Authors:** 


### EDITORIALS

The Business Office of the *Journal-Record* is 1 1-2 to 5 1-2 South Broad Street.
The Editorial Office is 1014-15 Century Building.
Address all Business Communications to *Journal-Record of Medicine*, 1 1-2 to 5 1-2 South Broad Street.
Make remittances payable *to The Journal-Record of Medicine.*

On matters pertaining to the Editorial and Original Communications, address Edgar G. Ballenger, M. D., Atlanta, Ga.
Reprints of Original Articles will be furnished at cost price. Requests for the same should always be made in THE MANUSCRIPT.

We will present, postpaid, on request, to each contributor of an original article, twenty (20) marked copies of *The Journal-Record of Medicine* containing such article.

### 'TWAS EVER THUS.

In the Atlanta Constitution, of Sept. 16th, we find this item of news from Macon, sent in, we presume, by their regular correspondent:

"At the meeting of the Board of Education to-day, Col. C. R. Pendleton, one of the members, proposed that a certain book listed for use in several of the schools be barred on account of two pages in it that are devoted to a treatise on the hookworm. He expressed a disbelief in the hookworm idea, and said that the children of the South should not be taught that they were being made lazy by the larvae of a worm entering their bodies through the soles of their feet, a feat which he believed was impossible. The board postponed action on the matter until the next meeting, but will make an investigation in the matter."

The gentleman referred to is editor of an influential daily paper, a man of parts, a trenchant writer, a popular citizen, but with it all in this instance he is an unreasoning obstructionist.

He doesn't believe in hookworm!

The researches of Stiles go for naught with him. The "could of witnesses" adduced by the Rockefeller commission bear no weight, and, with a lordly wave of his hand, he dismisses the whole subject as rot, unfit to be mentioned in the public schools.

How natural this sounds to the student of history!

Copernicus, who was unselfishly studying the movements of the solar system, and establishing great astronomical truths, was denounced by Martin Luther as an arrogant fool.

Gallileo, who proved his "three laws of motion," and removed physics from the realm of the occult, was tortured by an inquisition and forced to retract.

Harvey, who in the 17th century demonstrated the circulation of the blood, was for a while a most unpopular man—the butt of ridicule and the object of jealousy by his envious contemporaries.

Jenner, who demonstrated vaccination, and bequeathed to succeeding generations a priceless boon, who fought, tooth and nail, and there are still organized efforts (unfortunately fostered by those who should know better), to discredit his benificent and far-reaching discovery.

'Twas ever thus, and 'twill ever be.

No matter how wise and necessary some hygienic or prophylactic measure may be, or how important for the general welfare, some esteemed mossback or some venerable fossil will "rise to object," and will endeavor to place his nebulous notions in the scale with scientific facts.

Let our progressive friends in Macon be not discouraged. It is simply a case of history repeating itself.

G. M. N.

---

### THE FEE DIVISION EVIL AND PEARSON'S MAGAZINE.

On several occasions the Journal-Record of Medicine has published articles upon the subject of splitting fees and it may now be said to be one of the most important subjects for the medical profession to solve. The September issue of Pearson's magazine contains a sensational account of the evil in which is contained many truthful facts which will set the lay public to thinking. Whether this will do good or harm we will not venture to assert, but we hope it may check the spread of this evil which seems to be limited largely to the West and Middle West.

We are glad to say that the custom is one not yet widespread in thh South. It is probably not so frequent in the West as Pearson's leads the reader to believe, although it is certainly a matter of grave importance and one likely to grow worse unless checked.

The writer in Pearson's has an incorrect idea that "medical ethics" are to blame for this evil which, in fact, medical ethics has tended to lessen. He well says, however, that the trouble lies largely in the way the doctors are paid—the big fees going to the surgeon and specialist while the general practitioner everywhere is underpaid. Let him do more deliberate and careful work and demand and collect more for it.

---

### DR. FLOYD W. McRAE ELECTED SECOND VICE-PRESIDENT OF AMERICAN MEDICAL ASSOCIATION.

Again Atlanta has good reason to congratulate herself upon the honors bestowed by the American Medical Association; last year Dr. Dunbar Roy was elected chairman of the section on ophthalmology and otology and this year at the Los Angeles Convention Dr. Floyd W. McRae was honored by his election as Second Vice-President.

We take this opportunity to congratulate Dr. McRae on the reception of this distinct honor which he so well deserves both as a reward for his interest in the Association and regular attendance as well as for the eminence he has attained as a surgeon with more than a local reputation. We may well congratulate the Association upon its judgment in selecting such men thus to honor.

